# Cytokines Produced by Lymphocytes in the Incompetent Great Saphenous Vein

**DOI:** 10.1155/2018/7161346

**Published:** 2018-06-14

**Authors:** Ewa Grudzińska, Andrzej Lekstan, Ewelina Szliszka, Zenon P. Czuba

**Affiliations:** ^1^Department of Microbiology and Immunology, School of Medicine with the Division of Dentistry in Zabrze, Medical University of Silesia in Katowice, Jordana 19, 41-808 Zabrze, Poland; ^2^Department of General, Vascular, and Transplant Surgery, Medical University of Silesia in Katowice, Francuska 20-24, 40-027 Katowice, Poland

## Abstract

The pathogenesis of chronic venous disease (CVD) remains unclear, but lately inflammation is suggested to have an important role in its development. This study is aimed at assessing cytokines released by lymphocytes in patients with great saphenous vein (GSV) incompetence. In 34 patients exhibiting oscillatory flow (reflux) in GSV, blood was derived from the cubital vein and from the incompetent sapheno-femoral junction. In 12 healthy controls, blood was derived from the cubital vein. Lymphocyte culture with and without stimulation by phytohemagglutinin (PHA) was performed. Interleukins (IL) 1*β*, 2, 4, 10, 12 (p70), and 17A; interleukin 1 receptor *α* (IL-1ra); tumor necrosis factor-*α* (TNF-*α*); interferon-gamma (IFN-*γ*); and RANTES were assessed in culture supernatants by the Bio-Plex assay. In both stimulated and unstimulated samples, in the examined group, IL-1*β* and IFN-*γ* had higher concentrations and RANTES had lower concentrations when compared to those in the control group. In the examined group, IL-4 and IL-17A had higher concentrations without stimulation and TNF-*α* had higher concentrations with stimulation. The GSV samples had higher IL-2, IL-4, IL-12 (p70), and IFN-*γ* concentrations without stimulation and lower IL-2 and TNF-*α* concentrations with stimulation when compared to those of the upper limb in the examined group. These observations indicate that the oscillatory flow present in incompetent veins causes changes in the cytokine production by lymphocytes, promoting a proinflammatory profile. However, the relations between immunological cells, cytokines, and the endothelium require more insight.

## 1. Introduction

Chronic venous disease (CVD) is an extremely prevalent disease, especially in industrial countries. According to multiple studies, various stages of CVD may affect up to 85% of the population and more advanced clinical changes (C3–C6 in the Clinical-Etiology-Anatomy-Pathophysiology (CEAP) classification) occur in about 30% of the population [1–3]. Venous hypertension, incompetence of venous valves, and blood reflux are well described as mechanisms of venous disease, as well as the underlying changes in venous wall architecture [4, 5]. It is generally agreed that the inflammatory process, an established potent factor in vascular diseases, plays a pivotal role in the pathogenesis of CVD [6–9]. Recent research indicates that the oscillatory flow present in incompetent veins is a main factor leading to proinflammatory cytokine release by endothelial cells and may initiate and maintain leukocyte-mediated inflammatory reactions [10–12]. The mechanism of this process probably involves glycocalyx destruction by mechanical stress, which leads to increased expression of multiple leukocyte-recruiting factors like monocyte chemoattractant protein-1, macrophage inflammatory protein-1*β*, vascular cell adhesion molecule-1, and intercellular adhesion molecule-1 (ICAM-1) [13]. Next, endothelium-leukocyte interactions activate the inflammatory cascade [13].

However, there are very few studies concerning cytokines in this disease [14]. There are two interesting papers presenting the profile of 31 cytokines in plasma of CVD patients [10, 15], but the role of lymphocytes in CVD is obscure. Unlike neutrophils, which are almost absent probably because of the “leukocyte trap” mechanism, lymphocytes are found in normal numbers in varicose veins [8, 16]. It is postulated that they have a crucial role in venous ulcer development [17]. We hypothesized that due to the lymphocyte ability to roll along the venous wall and the contact with the endothelium pathologically changed by reflux, the lymphocytes in incompetent veins may present significant differences in the cytokine profile and in the reactivity to stimulatory agents in comparison to those in the healthy veins with laminar flow. Therefore, our study was designed to establish the release of cytokines by lymphocytes in CVD. Firstly, a difference in cytokine concentrations between the samples from healthy subjects and CVD patients was expected. Secondly, in the CVD group, the cytokine production may be different in the incompetent vein with oscillatory flow when compared to that in the same patients' healthy cubital vein with laminar flow. Finally, due to the contact with the pathologically changed endothelium, the lymphocytes may react differently to stimulating agents than the lymphocytes in healthy veins.

## 2. Materials and Methods

The study has been carried out in accordance with the Declaration of Helsinki and approved by the Bioethical Committee of the Medical University of Silesia (KNW/0022/KB1/31/I/12). All participants gave their written informed consent for the study.

The examined group consisted of 34 primary CVD patients with great saphenous vein (GSV) incompetence confirmed by Doppler ultrasound examination. The reflux at the sapheno-femoral junction (reflux time > 0.5 s) was confirmed in all patients in standing position, with blood flow elicited by manual squeezing. The control group included 12 volunteers with healthy GSV confirmed by Doppler ultrasound. The exclusion criteria involved history of venous thrombosis, pregnancy, diabetes, any inflammatory diseases present in the last two weeks, alcohol abuse, smoking, ulceration on the examined limb during the last month, and intake of anti-inflammatory drugs during the last two weeks.

Blood samples were obtained from the cubital vein in both groups and collected to vials containing heparin (10 IU/ml of blood). The patients from the examined group then underwent a standard surgical procedure of GSV stripping, with femoral nerve block and additional local anaesthesia. After inguinal incision and visualization of the GSV, the blood sample was obtained from the GSV directly below the incompetent sapheno-femoral junction into a heparinized vial. All samples were immediately transferred to the laboratory, maintaining 37°C. Cultures of lymphocytes were prepared either with lymphocyte-stimulating phytohemagglutinin (PHA) or with medium.

The lymphocytes were separated by the use of Histopaque gradients (1.119 g/ml and 1.077 g/ml). After centrifugation (700 ×*g*, 30 min), the separated lymphocytes were transferred to another vial and washed twice with phosphate-buffered saline (PBS) (250 ×*g*, 10 min). Microscopic morphological assessment of the cell population was performed, and no differences were found between groups. No significant contamination by other cells was found in the samples. A suspension of 2 mln lymphocyte cells/ml of medium (RPMI (Roswell Park Memorial Institute) 1640, 10% bovine serum, penicillin 100 *μ*g/ml, and 100 *μ*g/ml streptomycin). 0.5 ml of this suspension was added to 0.5 ml of PHA solution (20 *μ*g PHA/ml of medium) and to 0.5 ml of medium for no-stimulation samples. These suspensions were incubated for 24 h at 37°C, 5% CO_2_ atmosphere, and 99% humidity. After incubation and centrifugation (250 ×*g*, 10 min), the supernatant was collected to Eppendorf vials and stored at −80°C.

Interleukins 1*β*, 2, 4, 10, 12 (p70), and 17A (IL-1*β*, IL-4, IL-10, IL-12 (p70), and IL-17A); interleukin 1 receptor *α* (IL-1ra); tumor necrosis factor-*α* (TNF-*α*); interferon-gamma (IFN-*γ*); and RANTES were assessed. The samples were thawed directly before the Bio-Plex assay. The assay uses magnetic beads with anticytokine immunoglobulins to assess concentrations of many cytokines simultaneously. The samples were processed following the manufacturer's instructions (Bio-Plex Pro™ Human Cytokine Assays, Bio-Rad Laboratories) and read using Bio-Plex Manager™ Software. The statistical analysis was performed with the use of STATISTICA 10.0 software. Because there was no normal distribution of the examined cytokine samples, nonparametric tests were applied (Mann–Whitney *U* Test, sign test, and Wilcoxon signed-rank test). The leukocyte count and lymphocyte percentage had normal distribution; therefore, Student's *t*-test was applied.

## 3. Results and Discussion

### 3.1. Results

The examined group consisted of 34 patients, 85% of which were women. The median age was 47 ± 25 (21–68). The patients belonged to clinical CEAP classes C2-C3, with 43% in C2 and 57% in C3. The control group consisted of 12 patients, 92% of which were women. The median age was 36 ± 27 (29–64). The white blood cell count was 5.6 × 10^3^/*μ*l (mean) (3.7–8.8 × 10^3^/*μ*l) in the examined group and 5.9 × 10^3^/*μ*l (4.6–7.5 × 10^3^/*μ*l) in the control group. The lymphocyte percentage was 39% (mean) (22%–47%) in the examined group and 36% (23%–45%) in the control group. There were no statistically significant differences between groups.

**Figure 1:**
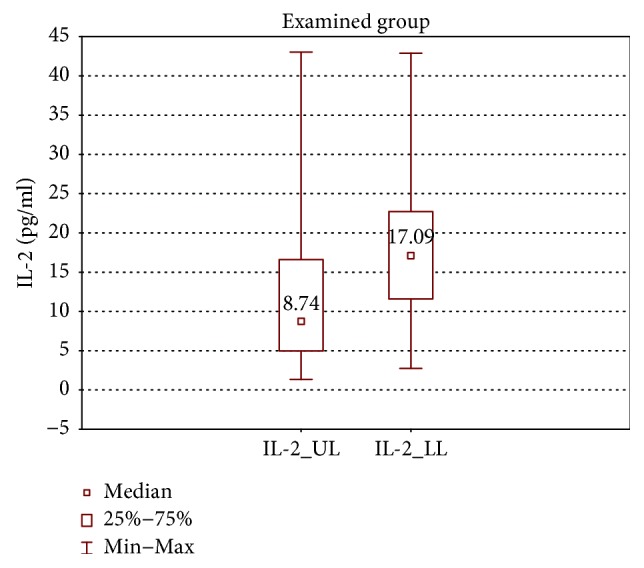
Comparison of IL-2 concentrations of the examined group in the upper (IL-2_UL) and lower limb samples (IL-2_LL), cultured without stimulation.

**Figure 2:**
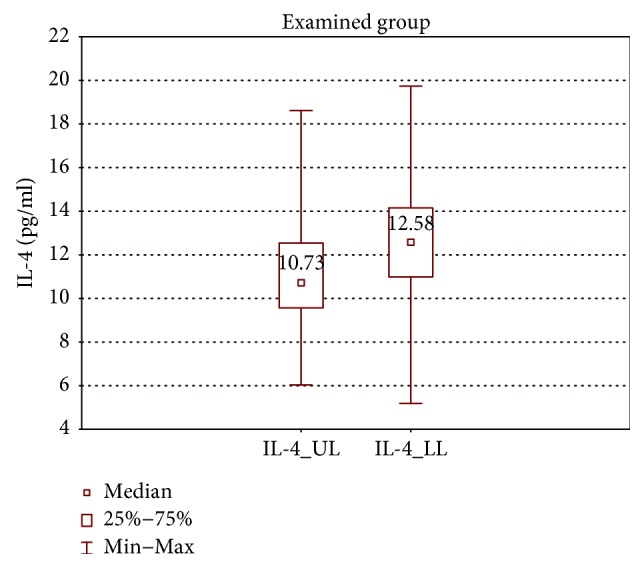
Comparison of IL-4 concentrations of the examined group in the upper (IL-4_UL) and lower limb samples (IL-4_LL), cultured without stimulation.

**Figure 3:**
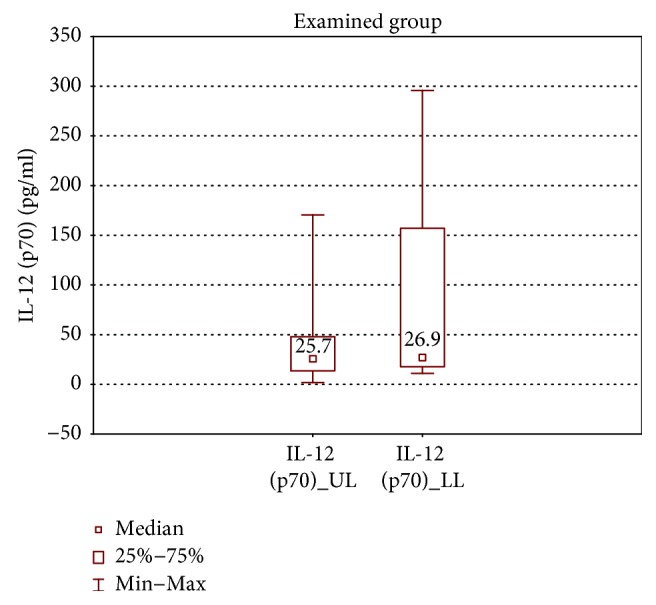
Comparison of IL-12 (p70) concentrations of the examined group in the upper (IL-12 (p70)_UL) and lower limb samples (IL-12 (p70)_LL), cultured without stimulation.

**Figure 4:**
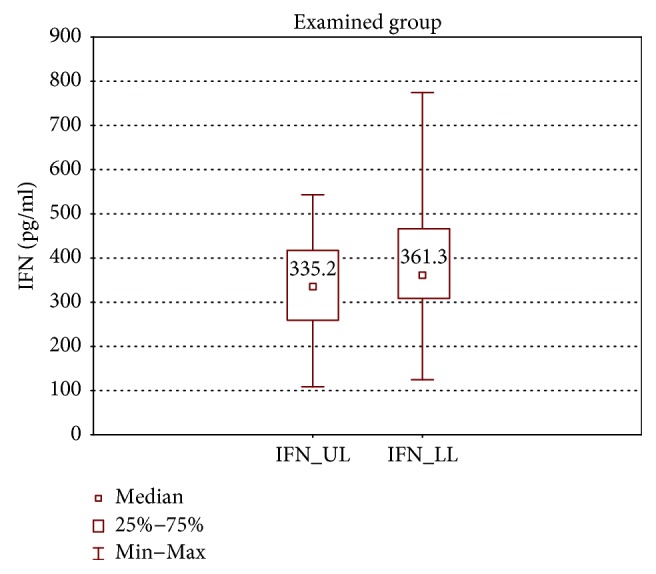
Comparison of IFN-*γ* concentrations of the examined group in the upper (IFN_UL) and lower limb samples (IFN_LL), cultured without stimulation.

In the samples cultured without stimulation of the examined group, significantly higher concentrations of IL-2, IL-4, IL-12 (p70), and IFN-*γ* were found in the incompetent GSV samples in comparison with the cubital vein samples of the same patients (expressed as median ± quartile deviation and range; IL-2: 17.1 ± 11.1 (2.7–42.9) pg/ml versus 8.7 ± 11.6 (1.3–43.0) pg/ml, *p* < 0.05; IL-4: 12.6 ± 3.2 (5.2–19.7) pg/ml versus 10.7 ± 2.9 (6.0–18.6) pg/ml, *p* < 0.01; IL-12 (p70): 26.9 ± 139.3 (11.1–295.8) pg/ml versus 25.7 ± 31.9 (1.9–170.5) pg/ml, *p* < 0.05; and IFN-*γ*: 361.3 ± 157.7 (124.7–774.3) pg/ml versus 335.2 ± 165.9 (108.6–543.1) pg/ml, *p* < 0.05). The above results are presented in Figures 1–4.

**Figure 5:**
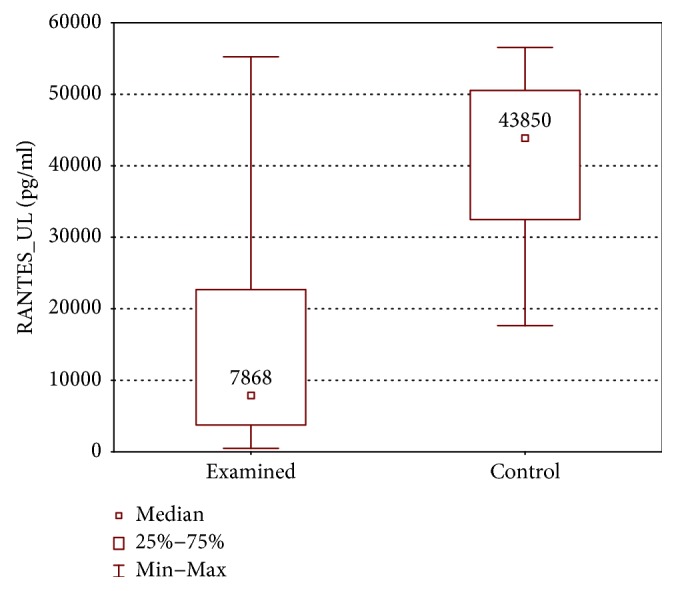
Comparison of RANTES concentrations between the upper limb samples of the examined and control group without stimulation (RANTES_UL).

When the upper limb samples cultured without stimulation were compared between groups, significantly higher concentrations of RANTES were found in the control group (RANTES: 43850 ± 18050 (17638–56553) pg/ml versus 7868 ± 18934 (486.3–55230) pg/ml, *p* < 0.01) (Figure 5).

**Figure 6:**
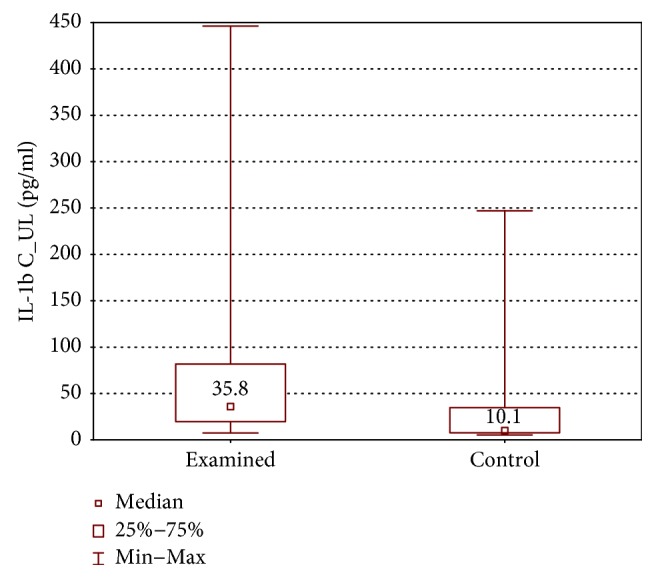
Comparison of IL-1*β* concentrations between the upper limb samples of the examined and control group without stimulation (IL-1*β*C_UL).

**Figure 7:**
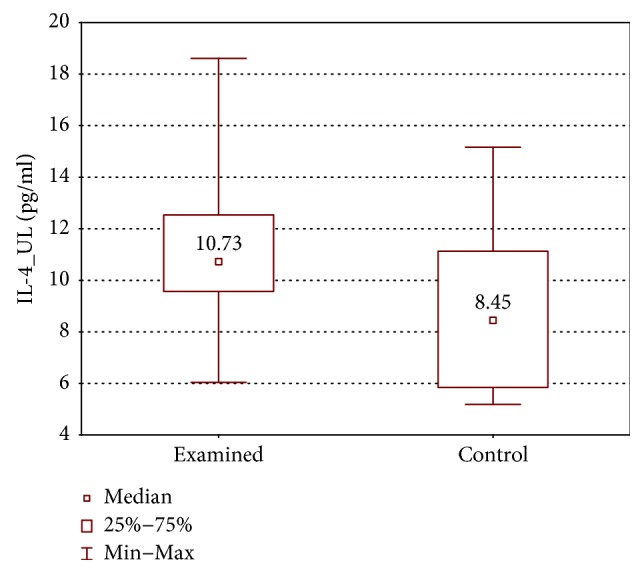
Comparison of IL-4 concentrations between the upper limb samples of the examined and control group without stimulation (IL-4_UL).

**Figure 8:**
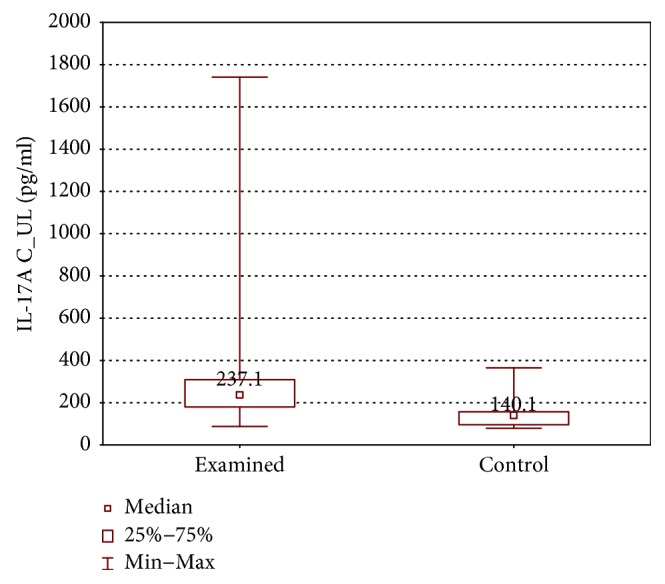
Comparison of IL-17A concentrations between the upper limb samples of the examined and control group without stimulation (IL-17AC_UL).

**Figure 9:**
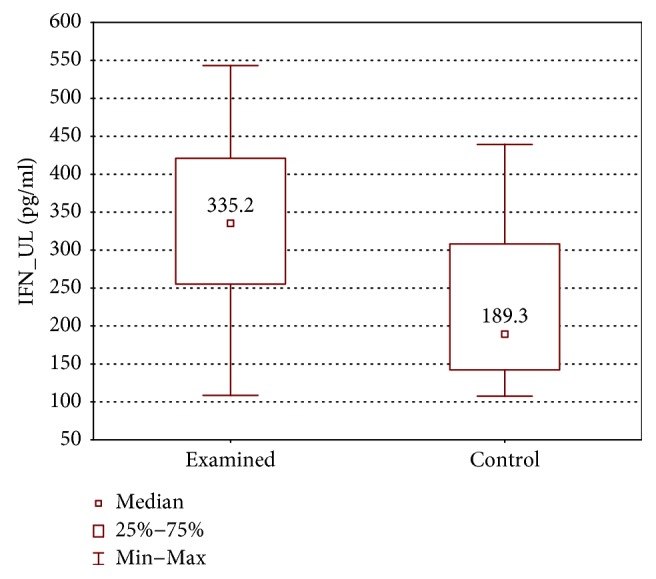
Comparison of IFN-*γ* concentrations between the upper limb samples of the examined and control group without stimulation (IFN_UL).

IL-1*β*, IL-4, IL-17, and IFN-*γ* concentrations were higher in the examined group (IL-1*β*: 35.8 ± 62.0 (7.5–446.3) pg/ml versus 10.1 ± 26.9 (5.3–247.0) pg/ml, *p* < 0.05; IL-4: 10.73 ± 2.97 (6.0–18.61) pg/ml versus 8.45 ± 5.28 (5.19–15.16) pg/ml, *p* < 0.05; IL-17A: 237.1 ± 107.0 (87–1740) pg/ml versus 140.1 ± 60.6 (79.3–365.2) pg/ml, *p* < 0.01; and IFN-*γ*: 335.2 ± 165.9 (108.6–543.1) pg/ml versus 189.3 ± 165.9 (107.4–439.3) pg/ml, *p* < 0.01) (Figures 6–9).

PHA stimulation resulted in significantly higher concentrations of all examined cytokines in both groups with the exception of IL-12 (p70) and RANTES. IL-12 (p70) had higher concentrations only in the upper limb of the examined group when compared to samples without stimulation, and RANTES concentrations were significantly higher only in the lower limb samples of the examined group. The upper limb samples of the examined group had higher RANTES concentrations with borderline statistical significance (7868 ± 18935 (486.3–55230) pg/ml versus 8394 ± 16724 (595.8–56391) pg/ml, *p* = 0.051).

**Figure 10:**
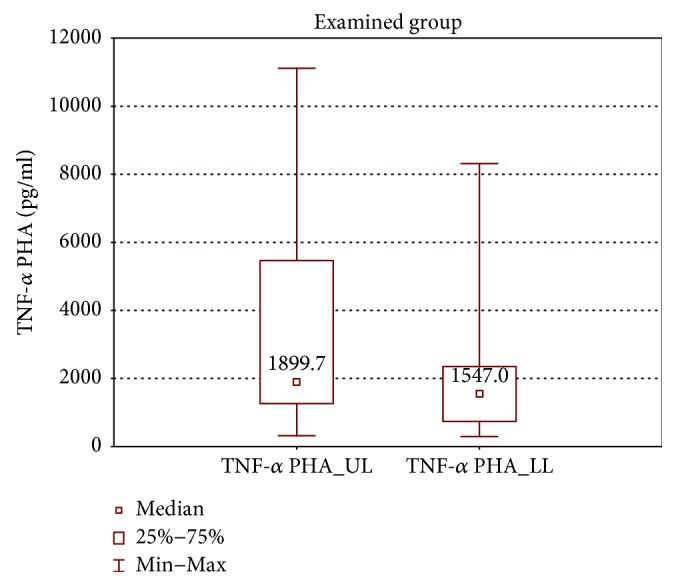
Comparison of TNF-*α* concentrations between the upper (TNF-*α* PHA_UL) and lower limb samples (TNF-*α* PHA_LL) of the examined group after PHA stimulation.

**Figure 11:**
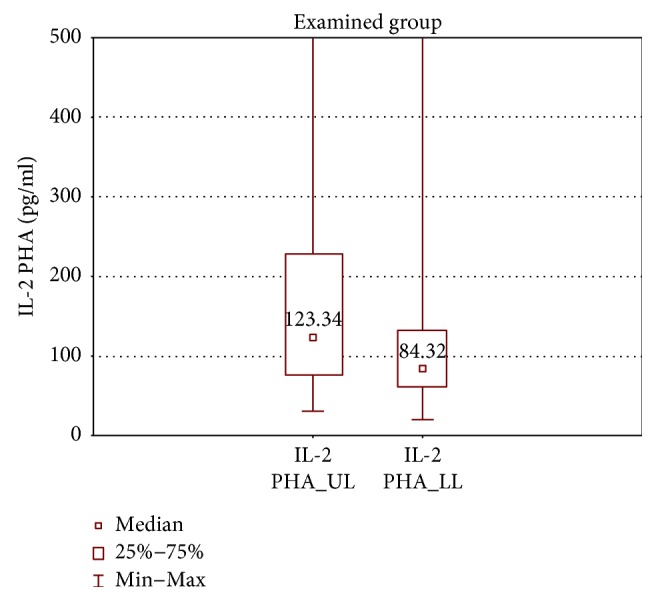
Comparison of IL-2 concentrations between the upper (IL-2 PHA_UL) and lower limb samples (IL-2 PHA_LL) of the examined group after PHA stimulation.

In the samples cultured with stimulation, the upper limb samples of the examined group had significantly higher TNF-*α* and IL-2 concentrations in comparison with the lower limb samples (TNF-*α*: 1899 ± 4204 (319.7–11115) pg/ml versus 1547 ± 1614 (294.4–8314) pg/ml, *p* < 0.01 and IL-2: 123.3 ± 152.3 (30.6–1805) pg/ml versus 84.3 ± 70.9 (20.0–3139) pg/ml, *p* < 0.01) (Figures 10 and 11).

**Figure 12:**
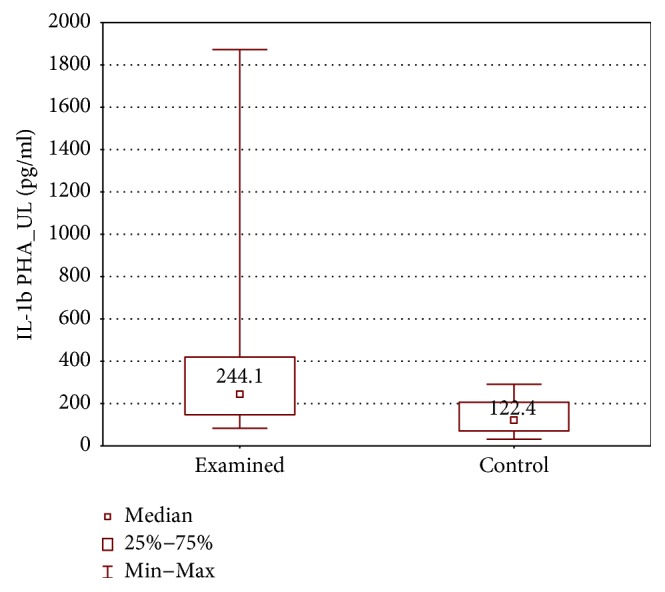
Comparison of IL-1*β* concentrations between the upper limb samples of the examined and control group, cultured with PHA stimulation (IL-1*β* PHA_UL).

**Figure 13:**
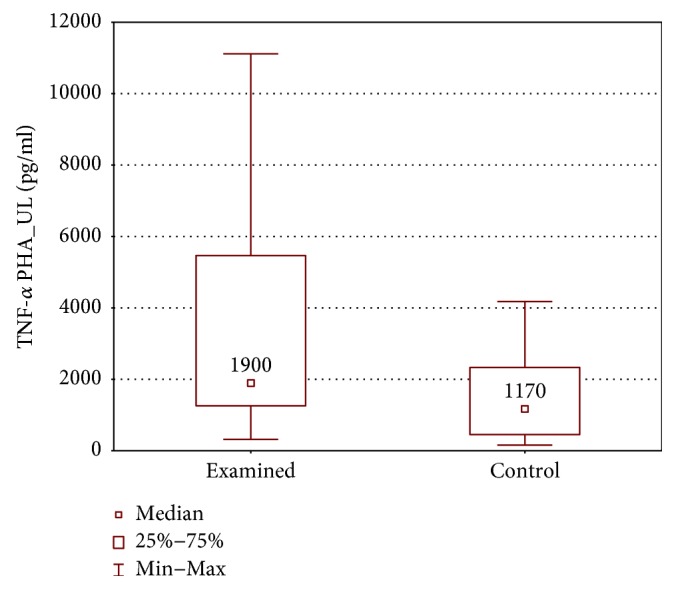
Comparison of TNF-*α* concentrations between the upper limb samples of the examined and control group, cultured with PHA stimulation (TNF-*α* PHA_UL).

**Figure 14:**
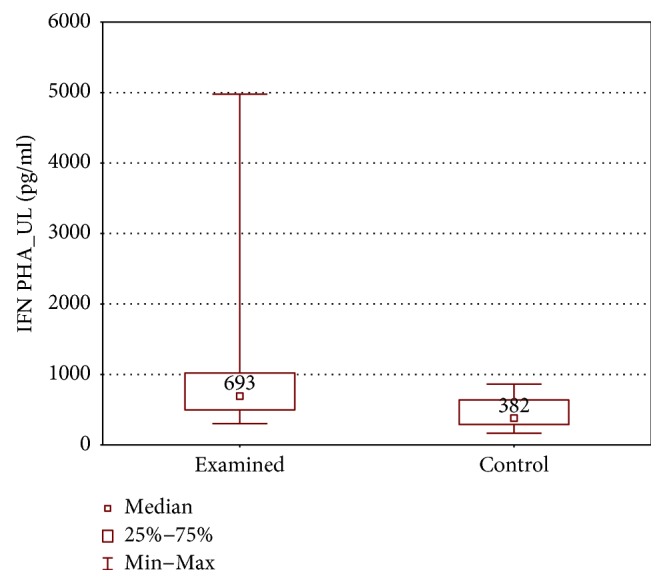
Comparison of IFN-*γ* concentrations between the upper limb samples of the examined and control group, cultured with PHA stimulation (IFN PHA_UL).

When the upper limb samples cultured with stimulation were compared between groups, higher IL-1*β*, TNF-*α*, and IFN-*γ* concentrations were found in the examined group (IL-1*β*: 244.0 ± 272.0 (83.6–1872) pg/ml versus 122.0 ± 135.5 (32.0–291.0) pg/ml, *p* < 0.01; TNF-*α*: 1900 ± 4204 (319.7–11115) pg/ml versus 1170 ± 1879 (159.0–4179) pg/ml, *p* < 0.05; and IFN-*γ*: 692.6 ± 523.9 (303.1–4978.26) pg/ml versus 382.3 ± 253.9 (165.0–587.2) pg/ml, *p* < 0.01) (Figures 12–14).

**Figure 15:**
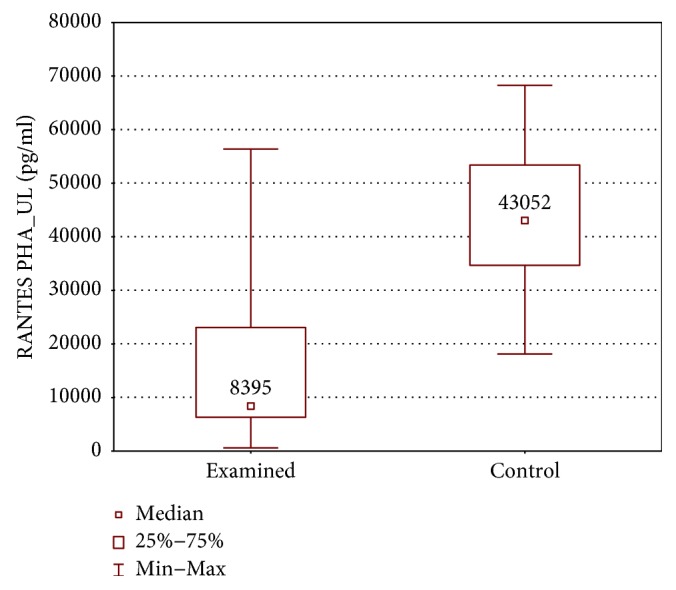
Comparison of RANTES concentrations between the upper limb samples of the examined and control group, cultured with PHA stimulation (RANTES PHA_UL).

RANTES had higher concentrations in controls (43052 ± 18701 (18124–68296) pg/ml versus 8395 ± 16725 (595.8–56392) pg/ml, *p* < 0.01) (Figure 15).

### 3.2. Discussion

One of our hypotheses was that due to the pathological blood flow present in the incompetent sapheno-femoral junction, causing endothelial damage, lymphocytes in contact with the venous wall may exhibit function changes. A study by Zamboni et al. examined 19 cytokines in CVD and showed that the flow correction caused significant amelioration of TNF-*α*, interferon-gamma-induced protein-10 (IP-10), IL-15, and granulocyte colony-stimulating factor (G-CSF) concentrations [10]. In another study, 31 cytokines/chemokines were examined in CVD patients before and after surgical hemodynamic correction. Before the procedure, 14 cytokines were increased in the examined group when compared to controls. After the laminar flow was restored, 11 cytokines were significantly reduced in the examined group compared to their basal levels [18]. This indicates that the turbulent blood flow is a major factor contributing to the inflammatory state in CVD. Indeed, we found significantly higher concentrations of IL-2, IL-4, IL-12 (p70), and IFN-*γ* in the samples derived from the incompetent sapheno-femoral junction in comparison to the upper limb veins, exhibiting laminar flow in the same patients. The only other two studies available, comparing varicose veins to general circulation of the same patients, showed that in varicose veins, there are elevated levels of cytokines: endothelial growth factor (EGF), platelet-derived growth factor (PDGF), RANTES, interleukin 6, interleukin 8 (IL-8), and monocyte chemoattractant protein-1 (MCP-1) [18, 19]. In our case unlike the other studies, the blood was drawn not from the GSV tributaries but from the incompetent sapheno-femoral junction. This assured that all samples were derived from the same anatomical region, where the sapheno-femoral valve incompetence occurs and the oscillatory flow is most evident. Blood derived from tributaries would inevitably come from various lower extremity regions, and therefore we found it less comparable. On the other hand, blood derived from the ankle or calf varices would undoubtedly be subject to more stasis and may have shown other differences, for example, in RANTES concentrations, which were elevated in varices in one of the studies [18]. Our results confirm the impact of reflux in the sapheno-femoral junction on the lymphocyte cytokine production in incompetent veins and suggest a tendency towards the proinflammatory state.

Higher concentrations of IL-1*β*, IL-4, IL-17A, and IFN-*γ* were found in cubital veins of CVD patients in comparison to the healthy group, further implying proinflammatory condition. However, a leukocyte-recruiting chemokine RANTES had significantly higher concentrations in the healthy group when compared to the varicose patients. In some of the few other available studies on cytokines in CVD, lower cytokine concentrations in varices were also found. Tisato et al. reported that reflux time correlates negatively with the expression of granulocyte-macrophage colony-stimulating factor (GM-CSF) [20], and Spath et al. observed that an increase in CVD symptoms in warmer months was associated with lower proinflammatory cytokine levels (eotaxin, IL-8, MCP-1, TNF-*α*, and vascular endothelial growth factor (VEGF)) [21]. On the other hand, in another study, elevated levels of proinflammatory markers CD31/platelet endothelial cell adhesion molecule (PECAM-1), CD46, and intercellular adhesion molecule-1 (ICAM-1) were found in the endothelium of varicose veins, with expression rising with the advancement of the disease [6]. There are also more inflammatory cells, especially monocytes/macrophages, found in varices mostly in the proximity of venous valves [8]. The elevated levels of cytokines in healthy veins or in veins after surgical correction may be partially explained by their role in repairing processes [10]; however, the equivocal results of the studies mentioned above along with our findings may suggest that the hypothesis of the proinflammatory state in CVD might require more insight into the role of cytokines/chemokines and into the interactions between the hemodynamics, endothelium, and immunological cells.

The PHA stimulation of lymphocytes was performed in our study in order to establish the potential of the lymphocytes in the incompetent veins to respond to activating factors. PHA is a known lymphocyte T stimulant. The lymphocyte B response to stimulation was therefore not assessed and requires further study, for example, with lipopolysaccharide as a stimulant.

Stimulation resulted in significantly higher IL-2 and TNF-*α* levels in the upper limb of the examined group when compared to the incompetent GSV. Our other observation, in which PHA caused a significant increase in IL-12 (p70) only in the upper limb of the examined group and not in the GSV samples or in the control group, may partially explain these results as IL-12 (p70) on its part stimulates lymphocyte T to produce TNF-*α*. The higher concentrations of TNF-*α* in the upper limb of the examined group were also higher in comparison to those of the control group, which requires explanation. In the literature concerning cytokines in CVD, we found that osteoprotegerin (OPG) is released in higher concentrations by endothelial cells in CVD patients and that it correlates positively with reflux time [6, 20]. OPG is an immunomodulating decoy receptor known to prevent activation of TNF-*α*, and its production is stimulated by estrogens. Taking into account that females are more prone to CVD and that in our study, the highest TNF-*α* concentrations were observed in the healthy veins of CVD patients in the stimulated cultures, TNF-*α* needs further examination as it might play a still unknown role in CVD pathogenesis.

Apart from TNF-*α*, in the examined group, higher IL-1*β* and IFN-*γ* levels were also found, confirming the earlier observation of elevated production of proinflammatory cytokines in incompetent veins. The production of proinflammatory cytokines in response to stimulation might be of significance, for example, in venous ulcers, where with the progression of venous stasis and vascular permeability, the lymphocytes are exposed to additional stimuli (chemokines and cytokines released by other leukocytes) [22].

We also observed that RANTES concentrations were significantly higher after PHA simulation only in the GSV samples. However, even without significant changes in RANTES concentrations in the control group after PHA stimulation, its concentrations remained significantly higher in comparison to those in the CVD patients. This might indicate lower lymphocyte potential to recruit other leukocytes in the varicose veins in response to stimulation.

In this study, we did not analyse the relation between cytokine concentrations and dilation of the venous wall. A significant reduction of contractile response to vasoactive agents was shown before, progressing with the severity of the disease, and, interestingly, angiotensin II affinity was significantly lower already in the early stages of the disease [23]. Cytokine concentrations in relevance to the severity of the disease and venous distension remain an interesting topic to investigate.

A small examined group is certainly a limitation of this study. However, the complexity of obtaining lymphocyte cultures involving immediate processing of each acquired sample and strict exclusion criteria prevent creating a large examined group. Similar obstacles were met by other researchers in this field [6, 18, 20].

## 4. Conclusions

Our observations indicate higher production of cytokines IL-2, IL-4, IL-12 (p70), and IFN-*γ* by lymphocytes derived from the incompetent sapheno-femoral junction, suggesting a role of reflux in lymphocyte function. High concentrations of IL-1*β*, IL-4, IL-17A, and IFN-*γ* in general circulation of CVD patients also show a proinflammatory tendency in comparison to those of the healthy group. However, RANTES is found in higher concentrations in healthy subjects than in CVD patients, and TNF-*α* and IL-2 concentrations are higher after stimulation in the healthy veins of the examined group. These observations require further studies of complex interactions between the cytokines, immunological cells, and endothelium subject to pathological blood flow in order to establish their role in CVD pathogenesis. Better understanding of the immunological aspects of this disease may lead to new preventive measures and more effective treatment, for example, with effective anti-inflammatory drugs.

## Data Availability

The Bio-Plex data used to support the findings of this study are available from the corresponding author upon request.
